# Environmental diel variation, parasite loads, and local population structuring of a mixed-mating mangrove fish

**DOI:** 10.1002/ece3.289

**Published:** 2012-07

**Authors:** Amy Ellison, Patricia Wright, D Scott Taylor, Chris Cooper, Kelly Regan, Suzie Currie, Sofia Consuegra

**Affiliations:** 1 IBERS, Aberystwyth UniversityPenglais Campus, Aberystwyth SY23 3DA, United Kingdom; 2 Department of Integrative Biology, University of GuelphGuelph, ON, N1G 2W1, Canada; 3 Brevard County Environmentally Endangered Lands ProgramMelbourne, Florida 32904; 4 Department of Biology, Mount Allison UniversitySackville, NB, E4L 1G7, Canada

**Keywords:** Environmental instability, genetic diversity, *Kryptolebias marmoratus*, outcrossing, parasites, selfing

## Abstract

Genetic variation within populations depends on population size, spatial structuring, and environmental variation, but is also influenced by mating system. Mangroves are some of the most productive and threatened ecosystems on earth and harbor a large proportion of species with mixed-mating (self-fertilization and outcrossing). Understanding population structuring in mixed-mating species is critical for conserving and managing these complex ecosystems. *Kryptolebias marmoratus* is a unique mixed-mating vertebrate inhabiting mangrove swamps under highly variable tidal regimes and environmental conditions. We hypothesized that geographical isolation and ecological pressures influence outcrossing rates and genetic diversity, and ultimately determine the local population structuring of *K. marmoratus*. By comparing genetic variation at 32 microsatellites, diel fluctuations of environmental parameters, and parasite loads among four locations with different degrees of isolation, we found significant differences in genetic diversity and genotypic composition but little evidence of isolation by distance. Locations also differed in environmental diel fluctuation and parasite composition. Our results suggest that mating system, influenced by environmental instability and parasites, underpins local population structuring of *K. marmoratus*. More generally, we discuss how the conservation of selfing species inhabiting mangroves and other biodiversity hotspots may benefit from knowledge of mating strategies and population structuring at small spatial scales.

## Introduction

Mangroves, together with coral reefs, are some of the most productive habitats of the planet and act as important nursery grounds and breeding sites for fish, crustaceans, and shellfish but also for birds, reptiles, and mammals of closely associated habitats (Alongi 2002; Duke et al. 2007). Around one-third of the world's mangrove forests have been lost over the past 50 years, mostly due to anthropogenic activities such as urban development, aquaculture, and overexploitation, with loss of biodiversity and habitat fragmentation being the largest threats to mangrove habitat persistence (Valiela et al. 2001; Alongi 2002). In addition, climate change will make many mangroves subject to the threat of sea-level rise. Rises of sea level are now recognized to be one of the main causes of predicted future losses in mangrove habitats (Gilman et al. 2008). Mangrove forests are home to many plants and animals with a mixed-mating system (the ability to self-fertilize and outcross) (Werner Jr 1967; Grahame 1969; Hsieh 1997; Klekowski 1998; Taylor 2000; Arnaud-Haond et al. 2006; Landry and Rathcke 2007) for which habitat loss and fragmentation can have consequences difficult to predict, given that genetic variation within populations is strongly influenced by mating system (Willi and Määttänen 2011) along with population size, spatial structuring, and habitat characteristics (Dionne et al. 2008; McCairns and Bernatchez 2008; Porlier et al. 2009).

In species that undergo partial self-fertilization (selfing), genetic differentiation between subpopulations can be more pronounced than in fully outcrossing species (Viard et al. 1996; Ingvarsson 2002; Charlesworth 2003). This is because selfing can increase homozygosity and create temporally stable genetic differences between habitats, mainly maintained by inbreeding (Viard et al. 1997; Charlesworth and Willis 2009). Selfing may also reduce the effective population size and contribute to decreased genetic diversity (Charlesworth et al. 1993; Jarne 1995) but has several advantages, namely transmission advantage (Fisher 1941), ability to reproduce when mates are few and far between (Darwin 1876) and increased colonization ability, as single dispersing individuals can establish new populations (Baker 1955). In mixed-mating species, strong founder effects and limited outcrossing could restrict the opportunities for the development of local adaptations (Barrett et al. 2008) although even small rates of outcrossing can introduce considerable genetic variation after only a few generations (Platt et al. 2010). Thus, selfing can be a major factor contributing to population structuring and local adaptation in both plants and animals.

The asymmetric distribution of selfing rates in animals, with high outcrossing rates being more frequently represented than high selfing rates, suggests that not only genetic factors (such as inbreeding depression; Escobar et al. 2011) but also ecological forces and mate availability play a crucial role in the evolution of mixed-mating in animals (Tsitrone et al. 2003; Jarne and Auld 2006; Auld 2010). Although most population genetics models are based on random mating (Wright 1978), even at small geographical scales, populations consist of subgroups of individuals with varying degrees of genetic diversity and relatedness (Carlsson et al. 1999; Coltman et al. 2003; Schweizer et al. 2007). This fine-scale nonrandom spatial distribution of individuals with regards to genotype and relatedness can influence population dynamics, for example, by influencing the rates of inbreeding or outbreeding (Chesser 1991).

**Figure 1:**
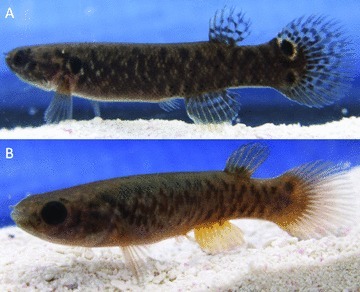
*Kryptolebias marmoratus* (A) hermaphrodite and (B) male.

The mangrove rivulus (*Kryptolebias marmoratus*, Fig. 1) is a unique selfing hermaphroditic vertebrate (Taylor 2000) and we used it as a model to investigate the potential interactions of mating system, dispersal, and local ecological pressures in shaping within-population genetic diversity and spatial structure of populations that do not fit the assumption of random mating. The species also represents the only vertebrate with an androdioecious mixed-mating reproductive strategy (with coexistence of males and selfing hermaphrodites) (Harrington 1961; Mackiewicz et al. 2006c) making it a unique model in evolutionary biology (Molloy and Gage 2006). *Kryptolebias marmoratus* is distributed between southern Florida/Yucatan and northern South America (29°N–0°N, 50°W–18°W) where it inhabits mangrove swamps under highly variable conditions of temperature, oxygen, salinity, and ammonia, mainly within the burrows of the land crabs, *Ucides cordatus* or *Cardisoma guanhumi* (Taylor 2000). Proportions of males typically vary between 1% and 25% and as such outcrossing rates are also variable among populations (Taylor 2001; Mackiewicz et al. 2006b). Thus, although in many populations of *K. marmoratus* highly homozygous selfing hermaphrodites predominate, those populations with the highest frequency of males display higher rates of outcrossing and more individual genetic diversity. This suggests that outcrossing happens mainly between males and hermaphrodites, although outcrossing between hermaphrodites cannot be completely ruled out (Mackiewicz et al. 2006b). Mangrove rivulus have developed unique adaptations to cope with environmental uncertainty, including the ability to live out of the water for prolonged periods (Taylor et al. 2008). The alternation of severe droughts with torrential rains and variable tidal regimes results in frequent changes in connectivity between rivulus populations, and this may have given rise to the evolution of mixed reproductive strategies. In these conditions, selfing would ensure reproduction during periods of isolation, whereas outcrossing would help to maintain genetic diversity when populations are reconnected (a “best-of-both-worlds” strategy; Mackiewicz et al. 2006c; Ellison et al. 2011). In addition to the wet–dry seasonal alternation, semidiurnal tides also exhibit a seasonal cycle, with extreme low tides in spring/summer and extreme high tides in fall. These tidal variations result in daily changes in environmental parameters such as temperature, exposure/submergence regimes, and nutrient availability (Lee and Joye 2006). *Kryptolebias marmoratus* may undergo long-distance dispersal and hybrids produced by outcrossing have been observed between local and very distant populations, suggesting that outcrossing and migration both play an important role in evolution of the species, although their relative importance remains unclear, particularly at the local scale (Tatarenkov et al. 2007). *Kryptolebias marmoratus* has been classed as “vulnerable” (http://www.natureserve.org) and listed as a “species of concern” (http://www.nmfs.noaa.gov/pr/species/concern) in North America due to mangrove habitat degradation.

We investigated the patterns of genetic diversity, migration rates, and population structuring of *K. marmoratus* in the mangrove forests of Calabash Caye, Belize (one of the most vulnerable regions to climate change of the world's mangroves, Alongi 2008) to test the hypothesis that geographical isolation and ecological pressures influence selfing/outcrossing rates and thus the genetic diversity and degree of population structuring at local scales. This population has a low percentage of males (2%) but relatively high levels of genetic diversity (Ellison et al. 2011). In addition, a previous study found that outbred individuals had higher heterozygosity and harbored lower parasite loads compared with their selfed counterparts, suggesting that parasite selective pressures may determine the rates of selfing and outcrossing (Ellison et al. 2011). Here, we compared the genetic composition and parasite loads of *K. marmoratus* at four locations with different degrees of spatial isolation and diel variation in environmental parameters. The expectation was that (1) more geographically isolated locations would display higher rates of genetic differentiation and lower values of genetic diversity and (2) ecological differences among locations (environmental diel fluctuation and parasite composition) may influence genetic diversity and rates of selfing/outcrossing. In particular, we expected that individuals from sites with more environmental fluctuation and higher parasite loads would display lower rates of selfing and more genetic diversity.

## Materials and Methods

### Sample collection

**Figure 2:**
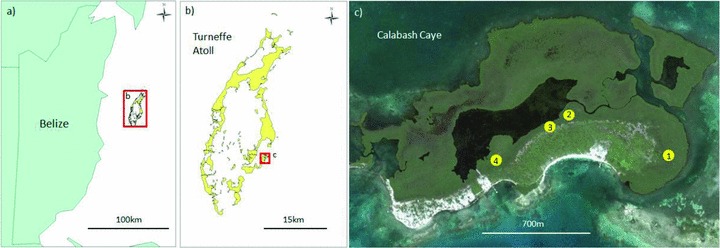
Four sampling locations of *Kryptolebias marmoratus* on Calabash Caye, Belize. (Photo courtesy of Google Earth).

**Figure 3:**
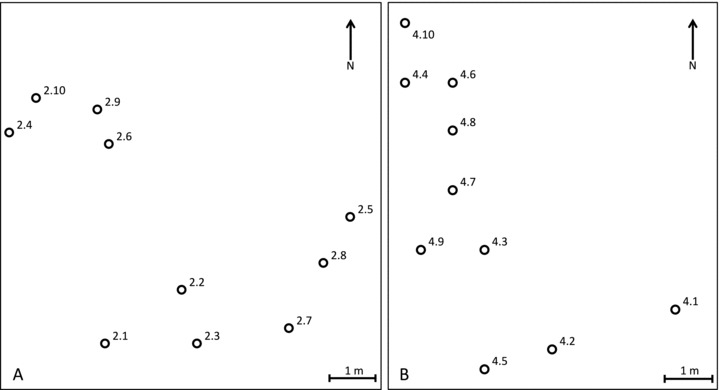
Relative positions of 10 crab burrows sampled in (a) site 2 and (b) site 4 for fine-scale structuring analyses.

Fish were collected over a period of three weeks in December 2009 at Calabash Caye, Turneffe Atoll, Belize (17°16′N, 87°48′W). A total of 118 fish (115 hermaphrodites and three males—one male in site 2 and two males in site 4; Table 1) were captured from temporary pools or the burrows of land crabs (*C. guanhumi*) by using cup traps and wire minnow traps (Mackiewicz et al. 2006b). Sampling was carried out at four locations on the island separated between 150 m and 1 km (Fig. 2). Locations were chosen based on their relative proximity or isolation from one another in order to provide insight at small scale population structure. Within two of the sampling locations (sites 2 and 4), the exact locations of 10 of the crab burrows occupied by fish were recorded for subsequent fine-scale structuring analyses (Fig. 3). The 10 burrows were separated between 1 to 16 m and were occupied by a total of 26 fish in site 2 and 19 in site 4. Fish were euthanized and stored in 95% ethanol. We included the data from a subset of 94 fish that had been previously screened for gill and gastro-intestinal parasites as described in Ellison et al. (2011).

**Table 1:** Genetic diversity (at 32 microsatellite loci) of *Kryptolebias marmoratus* at four sampling sites in Calabash Caye. Location (decimal lat-long), sample size (*N*); allelic richness (*Ar*); inbreeding coefficient (*F*_IS_ = (*H*_e_−*H*_o_)/*H*_e_); homozygosity by locus (*HL*); selfing rate from RMES (*S*_R_); selfing rate from INSTRUCT (*S*_I_); genetic admixture index (*J*′); average relatedness (*R*)

	Site 1	Site 2	Site 3	Site 4	All sites
Latitude	17.284	17.283	17.282	17.278	-
Longitude	−87.808	−87.814	−87.815	−87.815	-
*N*	8	57	18	35	118
Genetic diversity					
*Ar*	3.84	4.73	4.23	4.31	4.28
*F*_IS_	0.83	0.55	0.76	0.60	0.63
*HL*	0.86	0.68	0.87	0.73	0.77
*S*_R_	0.72	0.62	0.51	0.66	0.63
*S*_I_	0.76	0.62	0.68	0.69	0.69
*J*′	0.33	0.46	0.25	0.42	0.41
*R*	0.018	−0.001	0.013	0.007	0.000

### Diel variation in environmental parameters

In order to characterize the diel changes in environmental conditions of the mangrove rivulus habitat caused by tidal rhythms, water quality and depth was monitored in three crab burrows at each of the four field locations, over 24 h at 5:30, 11:30, 17:30, and 23:30 in December 2009. Diel variation in dissolved oxygen, temperature, ammonia, pH, salinity, and depth was used as a proxy for environmental instability of each sampling site. Water dissolved oxygen (%) and temperature were measured by placing a dual electrode (HQd field kit, Hach, Anachemia Science, Mississauga, ON, Canada) into the crab burrow approximately 6 cm below the water surface. A water sample (50–60 mL) was collected from the same location by carefully inserting a tube (6 cm) connected to a disposable syringe (60 mL) and withdrawing a sample for later determination of ammonia, pH, and salinity. Water samples were transferred to conical tubes (60 mL) and tubes were sealed immediately.

Water pH was determined immediately following return to the lab (∼30 min) with a pH electrode (Accumet combination pH electrode, Fisher Scientific, Markham, ON, Canada) and meter (Orion 520, Fisher Scientific). Salinity was determined with a portable refractometer (RHS-10ATC, Aqua Medic, China). Water was filtered (Whatman no. 1 filter paper) and frozen for later determination of ammonia levels (within 24–48 h). Total ammonia was measured on the water samples with the salicylate method (Verdouw et al. 1978). Absorbance was measured on a spectrophotometer (V-SPEC, Ocean Optics, Vernier, Beaverton, OR) at 650 nm.

### Microsatellite genotyping

Total genomic DNA was extracted from muscle tissue using the Wizard® SV 96 DNA Purification Kit (Promega Corp., Madison, WI, USA). DNA was quantified using the NanoDrop1000 v.3.7 Spectrophotometer (©2008 Thermo Fisher Scientific). Thirty-two of the 36 microsatellite loci described in Mackiewicz et al. (2006a) (excluding R6, R24, R36, and R45) were amplified in three different multiplex combinations. Polymerase chain reaction (PCR) mixes for each reaction contained 1X HotStarTaq Polymerase MasterMix (Qiagen® Multiplex PCR Kit), 0.2–6 μM of each primer, 2 μL DNA (concentrations between 5 and 20 ng/μL) and adjusted to the correct volume with ddH2O, following the protocol of Qiagen Multiplex PCR Kit. PCR cycling conditions were as followed: 15 min at 95°C (one cycle), touchdown PCR from 64°C to 56°C in steps of 2°C (two cycles per step and each cycle for 45 s at 95°C, 90 s at annealing temperature [*Tm*], and 90 s at 72°C), then 25 cycles at 56°C and finishing with 10 min at 72°C. PCR products were resolved on an ABI3130xl sequencer with GeneScan-500 LIZ size standard (Applied Biosystems CA) and analyzed using the software programme GeneMapper v 4.0.

### Genetic diversity, selfing rates, and relatedness

Loci were tested for linkage disequilibrium and Hardy–Weinberg equilibrium using GENEPOP (Raymond and Rousset 1995). Global genetic heterozygosity for each fish was estimated using the homozygosity by locus index (*HL*). This measure weighs contribution of each locus to overall homozygosity depending on its allelic variability. *HL* correlates better than uncorrected homozygosity (*H*_o_, measured as proportion of homozygous loci) with genome-wide homozygosity and inbreeding coefficients in open populations (Aparicio et al. 2006). From these values, mean homozygosity for each sampling site was estimated. Allelic richness per sample site was estimated using HP-Rare (Kalinowski 2005) for a minimum sample size of 8. The selfing rate at each site was estimated using the robust multilocus method of RMES (David et al. 2007). Pairwise estimates of relatedness were calculated using the Lynch and Ritland method (1999) in GENALEX v. 6, as recommended for high numbers of polymorphic loci (Van De Casteele et al. 2001). Mean relatedness values within each site and between all sites were estimated.

Analysis of variance (ANOVA) was used to test for differences in mean homozygosity, allelic richness, and relatedness among sites after the data were tested for normality. Homozygosity was arcsine transformed. Where overall significant differences were found, Tukey's post-hoc tests were used to identify significant pairwise comparisons.

### Population structuring

We conducted a spatial autocorrelation analysis to determine if the distribution of genotypes across the island and within the two sampling sites with the largest sample sizes (sites 2 and 4) was nonrandom. The autocorrelation coefficient *r* (Smouse and Peakall 1999) was calculated across all sites and within sites 2 and 4 using GenAlEx version 6 (Peakall and Smouse 2006). The significance of the autocorrelation was estimated by 999 permutations and 999 bootstrap replications were applied in order to determine the 95% confidence interval around *r*.

We also estimated the degree of genetic differentiation between sites (*F*_ST_) in FSTAT and the probability of genetic differences being significant was calculated based on 1000 permutations. The association between genetic distance (*F*_ST_) and the natural logarithm of geographic distance was assessed using a Mantel test (Hutchison and Templeton 1999). Mantel tests for associations of genetic and geographic distance within sites 2 and 4 were also carried out.

The Bayesian clustering method in INSTRUCT (Gao et al. 2007) was used to group fish into an optimal number of populations or selfing lines (*k*) based on their microsatellite genotypes. The most likely number of genetic groups was inferred from the largest rate of change in the likelihood function with respect to *k* (Δ*k*) as in STRUCTURE (Evanno et al. 2005). INSTRUCT was run 10 times at each value of *k* (1–15) with a burn-in of 10,000 replicates followed by 50,000 replicates to collect estimated parameters and likelihoods. The outputs of the repeated runs at each *k* were aligned using CLUMPP 1.1.1 (Jakobsson and Rosenberg 2007) and represented using DISTRUCT 1.1 (Rosenberg 2004). Posterior selfing rates of individuals, sites, and selfing lines were also calculated via INSTRUCT. A quantification of the likelihood of each individual to belong to each population represented by the *q*-value was used to assign individuals to populations or selfing lines. Under an admixed ancestry model, *q* represents the fraction of the genome of each individual inherited from ancestors in population *k* (Pritchard et al. 2000). Hybrids (individuals resulting from outcrossing between selfing lines) could thus be identified by having *q*-values intermediate between clusters (Vaha and Primmer 2006). A threshold *q*-value of 0.90 was used to identify selfed (inbred) individuals from those originating from recent outcrossing events (hybrids of different selfing lines) as in Ellison et al. (2011).

The extent of admixture in the genetic ancestry of each individual was estimated using a modification of Pielou's evenness index (*J*′) (Pielou 1966) based on the most likely group membership of each individual (*q*) derived from INSTRUCT. This index will range from 0 (if the genetic ancestry corresponds to a single group) to 1 (if it consists of equal proportion from different genetic groups). Differences in genetic diversity and admixture among lines were compared using ANOVA to test for differences in heterozygosity (*HL*), relatedness, and genetic admixture (*J*′).

In order to compare the distributions of the dominant genetic lines among the four sampling sites, individuals were classified to their major line of origin based on a *q*-value greater than 0.5 and individuals with *q*-values lower than 0.5 for all lines were deemed as “unclassified.” A *G*-test of independence was used to assess whether the distribution of lines among sampling sites was nonrandom (Logan 2010). William's correction for small sample sizes was applied. Significant excesses/deficits of lines at each site were determined by exploring the pattern of standardized residuals.

### Environmental instability and parasite loads

One-way ANOVA and Tukey's post-hoc pairwise comparisons were used to compare individual parasite loads among sampling sites. In order to assess the distribution of parasites among genetic lines, we used one-way ANOVAs followed by Tukey's post-hoc pairwise tests to compare individual parasite loads (from Ellison et al. 2011) of pure-bred individuals (*q* > 0.9) for each genetic line.

ANOVAs were also used to assess differences in environmental parameters (water temperature, depth, oxygen, ammonia, pH, and salinity) among sites. Environmental diel fluctuation at each sampling location was estimated as the variance for each variable. Significant differences in variances were tested using the Fligner–Killeen test of homogeneity of variances and 95% confidence intervals were calculated via bootstrapping (Efron 1982).

In order to assess the influence of both parasites and environmental diel variation in the variation of selfing rates, we first carried out a correlational PCA to analyze site differences in the three parasite infections and the three environmental variation factors analyzed. Regression analysis was then carried out to assess the association of selfing rate with the first two components of the PCA. This method allowed us to overcome potential problems derived from collinearity of some of the variables. All statistical analyses were carried out using R version 2.12.1.

## Results

### Genetic diversity, selfing rates, and relatedness

All loci deviated from Hardy–Weinberg equilibrium due to an excess of homozygotes (Table 1) and linkage disequilibrium was not detected between any pair of loci. Homozygosity was high as expected from a partially selfing species and values of *F*_IS_ oscillated between 0.55 and 0.83. Levels of homozygosity and selfing rates estimates indicated relatively frequent outcrossing events (Table 1). Sites differed significantly in mean homozygosity by locus (*F*_3,114_ = 4.17, *P* = 0.008) and mean relatedness (*F*_3,2368_ = 10.89, *P* < 0.001). Site 2 had significantly lower homozygosity (*HL* = 0.68 ± 0.24) than site 3 (*HL* = 0.87 ± 0.10; *S* = 0.88 ± 0.11). Site 2 also had significantly lower relatedness (−0.001 ± 0.038) than sites 3 (0.013 ± 0.062) and 4 (0.007 ± 0.040) while site 1 showed the highest average relatedness (0.016 ± 0.034; Table 1). In contrast, there were no significant differences in allelic richness among sites (*F*_3,124_ = 1.04, *P* = 0.377; Table 1). All the analyses were repeated excluding site 1 (*n* = 8) to account for the potential effect of the small sample size; the significance or direction of the results did not change with the exclusion of site 1.

### Population structuring

Spatial autocorrelation analyses indicated that the genotypes were not randomly distributed across the island (Fig. S1A). A significant positive correlation was found among individuals separated up to 200 m (*P* = 0.001) and significant negative correlations were found at 200–600 m (*P* < 0.001) and 800–1000 m (*P* = 0.002). However, we did not find any significant spatial autocorrelation among the burrows within site 2 (Fig. S1B) and only at 8–9 m within site 4 (*P* = 0.002), there was a significant positive correlation among genotypes (Fig. S1C).

Pairwise comparison of F*_ST_* values revealed significant genetic differences among all sampling locations (Table 2). Results from the Mantel test between genetic distance (*F*_ST_) and geographical distance indicated that there was no isolation by distance at the scale measured (*Z* = 228.47, *P* = 0.085). Mantel tests within sites 2 and 4 were also nonsignificant (site 2: *Z* = 71,516.25, *P* = 0.236; site 4: *Z* = 81,946.85, *P* = 0.169).

**Table 2:** Matrix of geographic distance (*m*, above diagonal) and genetic differentiation (*F*_ST_, below diagonal, asterisks indicate significance at *P < 0.01 and **P < 0.001) of sampling locations on Calabash Caye, Belize

Site	1	2	3	4
1	0	648	776	997
2	0.047*	0	153	562
3	0.075*	0.026**	0	442
4	0.063**	0.021**	0.028**	0

The Bayesian clustering in INSTRUCT found that the most likely number of selfing lines (*k*) was 5 (Figs. S2 and 4A), although the results must be considered approximate given that the method of Evanno et al. (2005) has not been extensively tested in selfing systems. The three only males sampled were classified as follows: the male from site 2 was purebred from line B (*q* > 0.9), the first male from site 4 was from line E (*q* = 0.8), and the second male from site 4 was classified as a hybrid (*q* < 0.5). Significant differences were found in the mean individual admixture index (*J*′) among sites (*F*_3114_ = 3.85, *P* = 0.011). The largest difference in admixture was found between site 2, with the highest value (*J*′ = 0.46 ± 0.25), and site 3 with the lowest (*J*′ = 0.25 ± 0.19) (Table 1).

**Figure 4:**
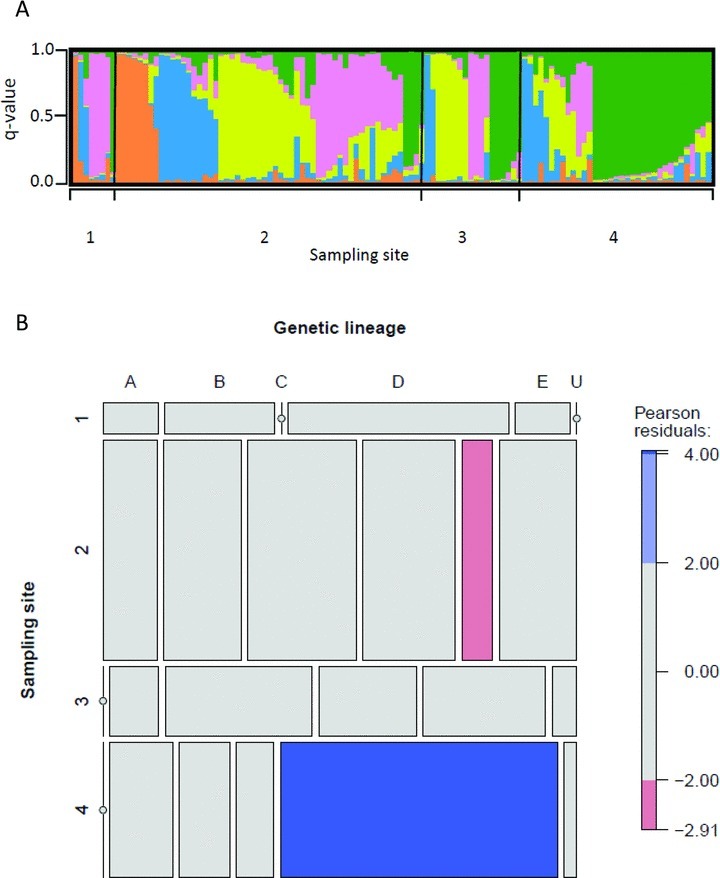
(A) Assignment of sampled *Kryptolebias marmoratus* to five self-fertilizing lines (clusters, A–E) using the Bayesian clustering algorithm INSTRUCT. Each individual is represented by a single bar and colors represent the individual's proportionate genetic membership (q) in a given cluster. (B) Mosaic plot representing significant excess (blue) or deficit (pink) of genetic line (A–E, U, unclassified) by sampling sites (1–4), based on standardized residuals. Size of rectangles is proportional to sample size.

### Diversity and distribution of selfing lines

Significant differences in mean heterozygosity (*HL*) were found among selfing lines (*F*_4113_ = 4.07, *P* = 0.004) (Table S1). Line C had the lowest *HL* (0.60) and line A had the highest (0.88). All lines had a mean relatedness value higher than the population average (range 0.0082–0.0538) and they significantly differed in relatedness (*F*_41497_ = 9.23, *P* < 0.001), line A being significantly more related than all other lines (Table S1). Lines also differed in their mean individual admixture index (*J*′) (*F*_4,113_ = 3.71, *P* = 0.007). The post-hoc Tukey's test revealed that line D had a significantly higher *J*′ (0.49 ± 0.26) than line E (0.31 ± 0.19) (Table S1). Proportions of genetic lines were found to be significantly associated with sampling site instead of randomly distributed (*G* = 49.26, *P* < 0.001). Differences were mainly due to site 2 having a significantly lower proportion of individuals assigned to line E than expected whereas site 4 had a significant excess of individuals of line E (Fig. 4B). The analysis was repeated excluding site 1 (*n* = 8) to account for the potential effect of the small sample size and the significance or direction of the results did not change (*G* = 42.05, *P* < 0.001).

### Environmental instability and parasite loads

Sampling sites differed in four of the six physical parameters measured. Site 1 had on average significantly higher water temperature (27.78 ± 1.39°C) than all the other sites (site 2 = 26.39 ± 0.57°C, site 3 = 25.90 ± 0.46°C, site 4 = 26.39 ± 0.61°C) (*F*_3,44_ = 10.96, *P* < 0.001) (Fig. S3). Sites 1 and 3 had significantly higher pH (site 1 = 7.41 ± 0.07, site 3 = 7.44 ± 0.11) than sites 2 and 4 (site 2 = 7.25 ± 0.14, site 4 = 7.22 ± 0.20) (*F*_3,44_ = 7.83, *P* < 0.001) (Fig. S3). Site 1 also had significantly higher salinity than site 4 (site 1 = 45.50 ± 0.80, site 4 = 38.67 ± 1.07) (*F*_3,44_ = 3.64, *P* = 0.020) (Fig. S3). Site 1 had on average a significantly greater average water depth (3.04 ± 2.68 cm) than all other sites (site 2 = 0.08 ± 0.20, site 3 = 0.46 ± 0.78, site 4 = 0.92 ± 1.02) (*F*_3,44_ = 9.44, *P* < 0.001). There were no significant differences in dissolved oxygen (*F*_3,44_ = 0.46, *P* = 0.713) and ammonia (*F*_3,44_ = 1.26, *P* = 0.298) among sites (Fig. S3).

**Figure 5:**
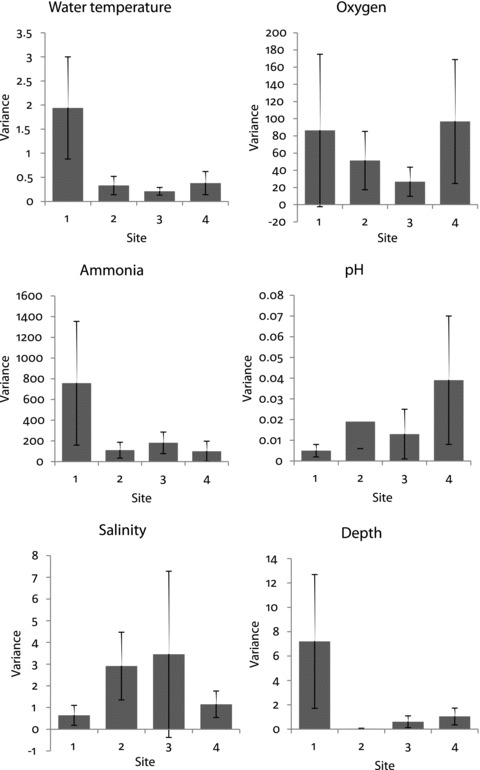
Diel variation in water temperature, oxygen, ammonia, pH, salinity, and depth at four sampling sites on Calabash Caye, Belize. Error bars represent 95% confidence intervals.

We used the variance of the physical measurements taken at four times covering a period of 24 h (diel variation) as a proxy for environmental instability and found significant differences among sites in the variation of three of the parameters measured, water temperature (χ^2^ = 8.02, *P* = 0.046), ammonia (χ^2^ = 10.02, *P* = 0.018), and depth (χ^2^ = 20.26, *P* < 0.001). Site 1 had significantly greater variation in water temperature (1.94 ± 1.06) than all other sites (site 2 = 0.33 ± 0.19, site 3 = 0.21 ± 0.08, site 4 = 0.38 ± 0.24) (Fig. 5). Variation in ammonia at site 1 (756.50 ± 597.43) was also significantly greater than at all other sites (site 2 = 110.16 ± 76.51, site 3 = 180.94 ± 104.27, site 4 = 99.91 ± 97.25) (Fig. 5). Site 1 had significantly greater variation in depth (7.20 ± 5.49) than all other sites (site 2 = 0.04 ± 0.04, site 3 = 0.61 ± 0.48, site 4 = 1.04 ± 0.69). No significant differences among sites were found in the variation of oxygen (χ^2^ = 3.30, *P* = 0.348), pH (χ^2^ = 3.54, *P* = 0.316), and salinity (χ^2^ = 5.32, *P* = 0.150). Taken together, these results indicated that site 1 had the highest diel variation of the four sampled locations in the environmental parameters measured.

The three most common infections in the fish analyzed were gastro-intestinal acanthocephalans, trichodinid protozoans, and bacterial gill cysts (Ellison et al. 2011). Sites differed significantly in the presence of two of these infections. Site 1 had a significantly higher average number of acanthocephalans than all other sites (*F*_3,90_ = 10.69, *P* < 0.001; Ellison et al. 2011) and site 4 had significantly higher average number of bacterial gill cysts than all other (*F*_3,90_ = 11.64, *P* < 0.001). No significant differences were found in trichodinid numbers among sites (*F*_3,90_ = 1.54, *P* = 0.209). No significant differences in acanthocephalan and trichodinid infections were found among genetic lines (acanthocephalans; *F*_4,33_ = 0.17, *P* = 0.952, trichodinids; *F*_4,33_ = 1.75, *P* = 0.163). However, differences in bacterial gill cysts among genetic lines were found to be significant (*F*_4,33_ = 4.15, *P* = 0.008) and line E had the highest occurrence of bacterial gill cysts, significantly higher than lines C and D.

**Figure 6:**
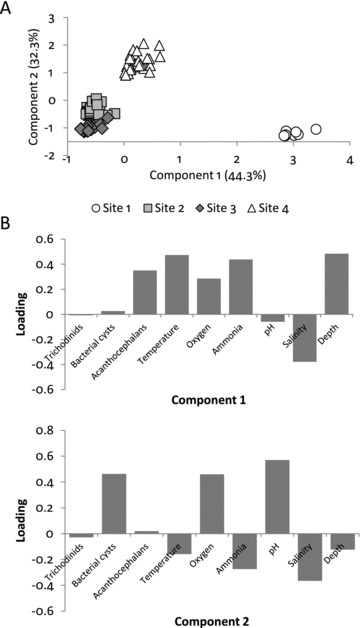
(A) Principle component analysis (PCA) of variation in parasite infections and environmental stability (variation in water temperature, oxygen, ammonia, pH, salinity, and depth) at four sampling sites on Calabash Caye, Belize. (B) Loadings of first two components from PCA of all parasite infections and environmental variation factors.

The first two components of the PCA of all parasite and environmental variation factors explained 76.6% (44.3% and 32.3%, respectively) of the variation among sites (Fig. 6A). The highest loadings on component 1 were depth (0.484), temperature (0.473), and ammonia variation (0.437) while pH (0.570), bacterial gill cysts (0.462), and oxygen variation (0.460) were the highest loadings on component 2 (Fig. 6B). Regression revealed a significant association between component 1 and selfing rate (*F*_2,91_ = 5.09, *P* = 0.026) but not for component 2 (*F*_2,91_ = 0.04, *P* = 0.833).

## Discussion

Our study highlights the importance of the mating system (likely influenced by environmental fluctuation and parasites) in the population structuring of a mixed-mating species. A mating system of selfing and occasional outcrossing may result in highly distinct genetic composition of populations even at very fine spatial scales. The uncertainty and harshness of the mangrove habitat, together with *K. marmoratus* colonizing ability, could help explaining the maintenance of mixed-mating on this species (Mackiewicz et al. 2006c). On this basis, we hypothesized that ecological pressures and geographical isolation would play a crucial role in the population structuring of *K. marmoratus*, by influencing selfing rates and consequently genetic diversity. Our results suggest that, at the local scale, selfing rates may be influenced to some extent by environmental diel variation and parasite selective pressures, thus shaping the population structure of *K. marmoratus*. In contrast, at this spatial scale, geographical distance does not seem to play an important role on population differentiation.

We found significant differences in homozygosity, relatedness, and degree of admixture among four locations sampled in Calabash Caye (Belize), separated between 150 m and 1 km. In addition, *F*_ST_ analyses identified significant genetic differences among all sampling locations and the composition of genetic lines varied significantly among sites. The autocorrelation analyses indicated that there was nonrandom association of genotypes at small spatial scales (200 m) but a negative correlation at greater distances and within sampling sites. The two sites with the highest differences in relatedness and homozygosity (sites 2 and 3) were the closest geographically and we did not find evidence of isolation by distance (albeit with a low number of comparisons), suggesting that differentiation could not be solely attributed to spatial isolation, as it has been often observed at localized scales (Bradbury et al. 2006; Barson et al. 2009). Thus, our results suggest that factors other than the distance, such as the mating system, small population size, and/or ecological selective pressures could play an important role in the genetic structuring of the species, as observed in mixed-mating plants (Willi and Määttänen 2011).

Spatial and temporal instability in environmental parameters can influence the mating system and help maintain intermediate rates of selfing in mixed-mating populations (Charbonnel et al. 2005). Outcrossing can facilitate the adaptation to novel environments, including host–parasite coevolution (Morran et al. 2011). Our four sampling locations differed significantly in parasite composition and environmental instability (diel variation). We found significant differences in individual parasite loads and genotype composition among sites, suggesting that parasite pressures might (at least partially) be related to the distribution of selfing lines among sites. The two sites with the highest incidence of two of the parasites analyzed (acanthocephalans in site 1 and bacterial cysts in site 4) were dominated by two different genetic lines (D in site 1 and E in site 4), although the relation was only significant for line E and bacterial cysts in site 4. We had previously shown a positive association between parasite loads, homozygosity and selfing in this species; outbred progeny being more genetically variable and carrying lower parasites loads than selfed progeny (Ellison et al. 2011). The spatial differences observed among parasite composition and selfing lines and the potential advantage conferred to outcrossed progeny by genetic diversity might suggest that (1) parasites could spread more slowly in genetically diverse host populations than in their genetically homogeneous counterparts and (2) parasites could be promoting local adaptation in *K. marmoratus* at small scales. Similar patterns have been observed in *Daphnia magna* and its parasite *Octosporea bayeri*, where parasites appear to be specialized on the local host clone community and immigrants have the advantage of being rare (Ebert 2008), or in the asexual snail *Potamopyrgus antipodarum* (Jokela et al. 2009), suggesting negative frequency dependent selection. The five distinct genetic lines identified in our study differed significantly in heterozygosity, relatedness, and degree of admixture, suggesting that particular lines could outcross more than others, although the low proportion of males prevented testing the hypothesis that the production of males was different among lines. Thus, the confirmation of this hypothesis warrants more experimental studies (Kawecki and Ebert 2004).

In mixed-mating populations, such as *K. marmoratus*, that display an array of selfing rates from extensive selfing to extensive outcrossing (Mackiewicz et al. 2006b), outcrossing can be selected for if it contributes to avoiding inbreeding depression and help to adapt more rapidly to environmental change (Anderson et al. 2010; Morran et al. 2011). In laboratory lines of the mixed-mating *Caenorhabditis elegans*, populations with the highest frequency of males and outcrossing are characterized by being genetically diverse following exposure to novel environmental conditions (Morran et al. 2009b). Moreover, the proportion of outcrossing can be increased by exposure to stress (Morran et al. 2009a). Variable exposure to environmental pressures could, thus, be responsible for different rates of outcrossing observed in natural mixed-mating populations (Anderson et al. 2010).

Habitat fragmentation and obstruction of tidal flows within mangrove swamps can lead to sediment accumulation, lower water depths, extreme salinity, and low dissolved oxygen (Layman et al. 2004). Changes in water chemistry are known to affect the behavior and physiology of *K. marmoratus*. For example, low oxygen induces *K. marmoratus* to emerse (Regan et al. 2011) what in turn alters metabolism (Ong et al. 2007), nitrogen excretion (Frick and Wright 2002; Litwiller et al. 2006), gene expression (Hung et al. 2007), and gill morphology (Ong et al. 2007). In addition, variation in salinity affects ion and water transport, ionocyte size and number, nitrogen excretion, and amino acid levels (Frick and Wright 2002; Leblanc et al. 2010). Loss of hydrological connectivity caused by habitat fragmentation can also affect prey consumption and parasite resistance in estuarine fish, which will likely have fitness consequences (Rypel and Layman 2008). Our results suggest that selfing rates might be influenced by both environmental (diel) instability and parasite pressures. However, the results must be interpreted with caution given the sample size and type of sampling. Seasonal variation in tidal changes has been previously observed in Belize mangroves resulting in local differences in environmental parameters (e.g., in dissolved oxygen and nitrogen fixation) (Farnsworth and Ellison 1996; Lee and Joye 2006). Although the exclusion of the location with the smallest sample size (site 1) from the analyses did not change the significance or direction of the differences in relatedness and genetic diversity among sites, the nature of our environmental sampling (diel variation) prevented us to take into account seasonal variation in tidal regimes. Therefore, further temporal and spatial comparisons at different scales would help to clarify the role of environmental instability in the population structuring of *K. marmoratus*.

Reductions in population density influence selfing rates (Chen 2000) and the ability to self-fertilize confers several advantages in harsh environmental conditions, such as reproductive assurance (Baker 1955). However, our results suggest that for the mangrove rivulus, and most likely for other animal selfing species, an important effect of habitat loss could be the extirpation of unique genetic lineages, reducing the genetic diversity and adaptive potential of the species.

Reduction in connectivity between marine and inland ecosystems due to habitat fragmentation represents a pervasive threat to tropical mangrove estuaries, and the consequences will likely become further exacerbated by climate change and predicted rises in sea level (Alongi 2008). Island-dominated mangroves, such as those in Belize, are particularly sensitive to changes in sea level since they are largely isolated from the influence of terrestrial sediments (McKee et al. 2007). Our results suggest that mangrove conditions influence the mating system of *K. marmoratus* at surprisingly small spatial scales, which in turn influence genetic structure. Given the abundance of plant and animal mixed-mating species in mangrove ecosystems (Werner Jr 1967; Grahame 1969; Hsieh 1997; Klekowski 1998; Arnaud-Haond et al. 2006; Landry and Rathcke 2007), understanding those factors affecting population structuring and distribution of genetic diversity in mangrove selfing species appears critical for implementing efficient conservation programmes.

## Supporting Information

**Table S1.** Genetic diversity and relatedness of five selfing lines (A–E) of *Kryptolebias marmoratus* on Calabash Caye identified by Bayesian clustering based on 32 microsatellite loci.

**Figure S1.** Results of spatial autocorrelation analysis based on genotypes of 32 microsatellite loci of *Kryptolebias marmoratus* from (A) across all sampling locations, (B) site 2, and (C) site 4.

**Figure S2**. Likelihood plots for the identification of the optimal number of populations or lineages (*k*) in INSTRUCT following (Evanno et al. 2005).

**Figure S3.** Mean (±1 SE) water temperature, percentage of oxygen, ammonia, pH, salinity, and water depth of four sampling sites at Calabash Caye, Belize.
