# Supplementary material for: Environmental diel variation, parasite loads, and local population structuring of a mixed-mating mangrove fish
Source: Ecol Evol. 2012 Jul;2(7):1682–95. doi: 10.1002/ece3.289 (PMC3434939; doi:10.1002/ece3.289)
Supplement: Supplementary file 1 [file ece30002-1682-SD1.doc]

**Table S1.** Genetic diversity and relatedness of 5 selfing lines (A-E) of *Kryptolebias marmoratus* on Calabash Caye identified by Bayesian clustering based on 32 microsatellite loci. Sample size (*N*); homozygosity by locus (*HL*); relatedness (*R*); genetic admixture index (*J’*).

|  | **A** | **B** | **C** | **D** | **E** |
| --- | --- | --- | --- | --- | --- |
| *N* | 8 | 18 | 29 | 23 | 32 |
| *HL* | 0.81 | 0.67 | 0.84 | 0.71 | 0.75 |
| *R* | 0.054 | 0.013 | 0.008 | 0.010 | 0.015 |
| *J’* | 0.26 | 0.46 | 0.45 | 0.49 | 0.31 |


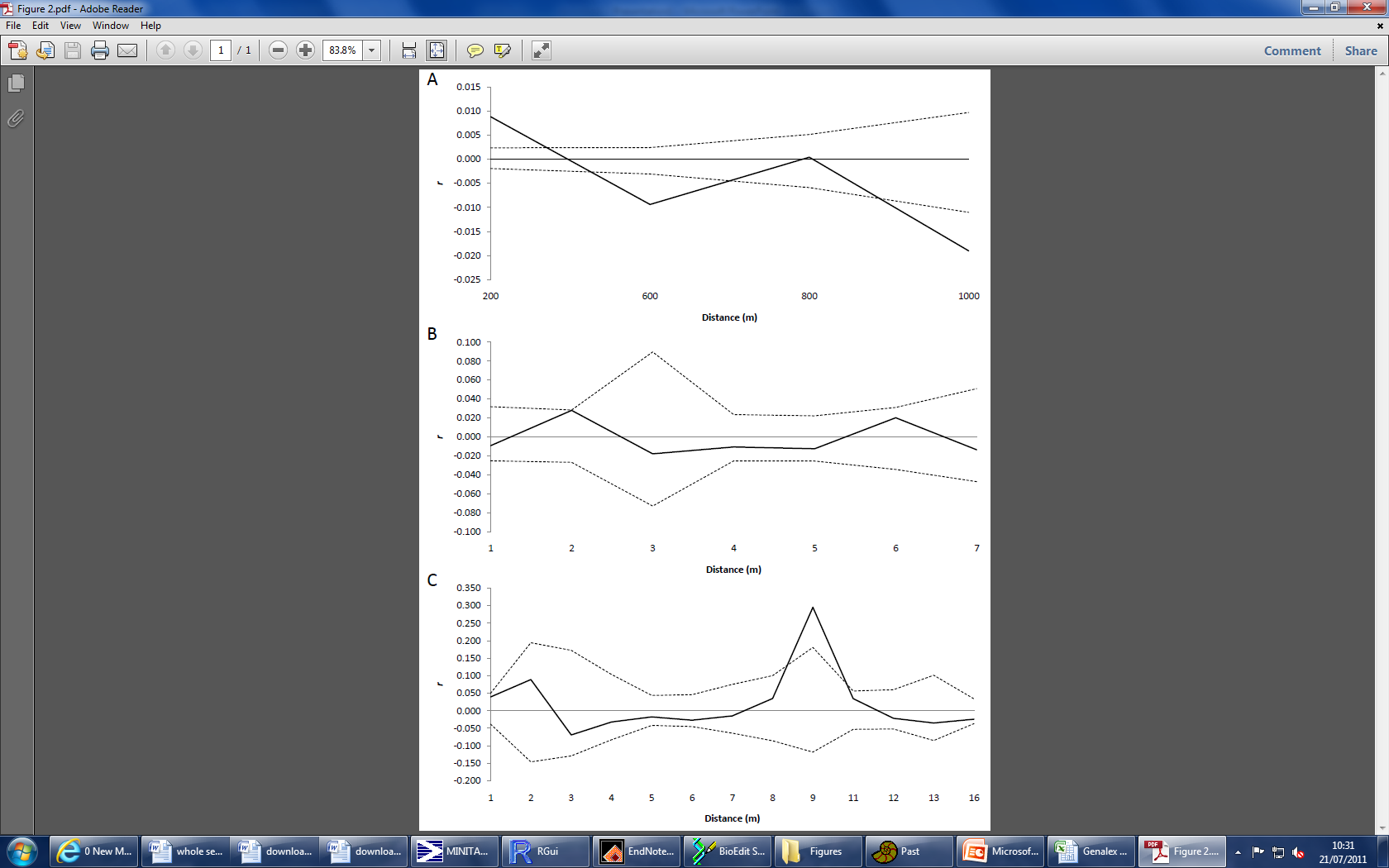


**Figure S1.** Results of spatial autocorrelation analysis based on genotypes of 32 microsatellite loci of *Kryptolebias marmoratus* from a) across all sampling locations, b) site 2 and c) site 4. Dashed lines represent 95% confidence intervals associated with null hypothesis of no spatial structure (i.e. *r* = 0). A significant association is observed among individuals within 200 m of distance (P = 0.001) whereas significant negative correlations are observed between 200-600 m (P <0.001) and 800-1000 m (P = 0.002).

**
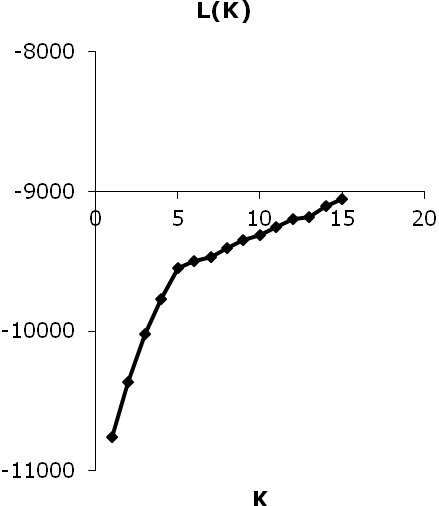
** **
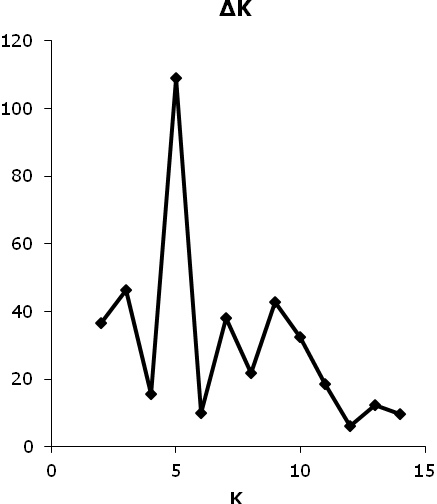
**
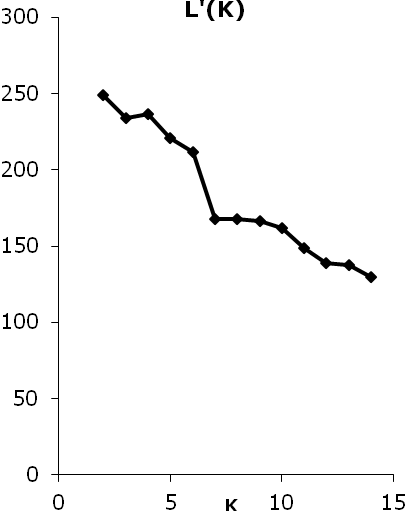

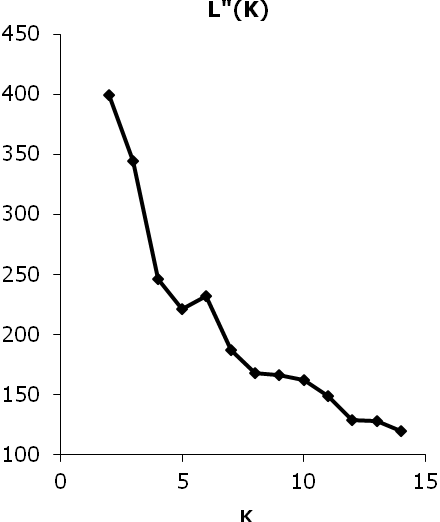


**Figure S2**. Likelihood plots for the identification of the optimal number of populations or lineages (k) in INSTRUCT following (Evanno et al. 2005). **L(K)**: average likelihood of each k value over 10 runs in INSRUCT, **∆K**: difference of likelihood values of each k, **L’(K)**: rate of change of the likelihood distribution, **L’’(K)**: absolute values of the second order rate of change of the likelihood distribution. The estimated number of populations in the *Kryptolebias marmoratus* samples from Calabash Caye (Belize) was 5.

**
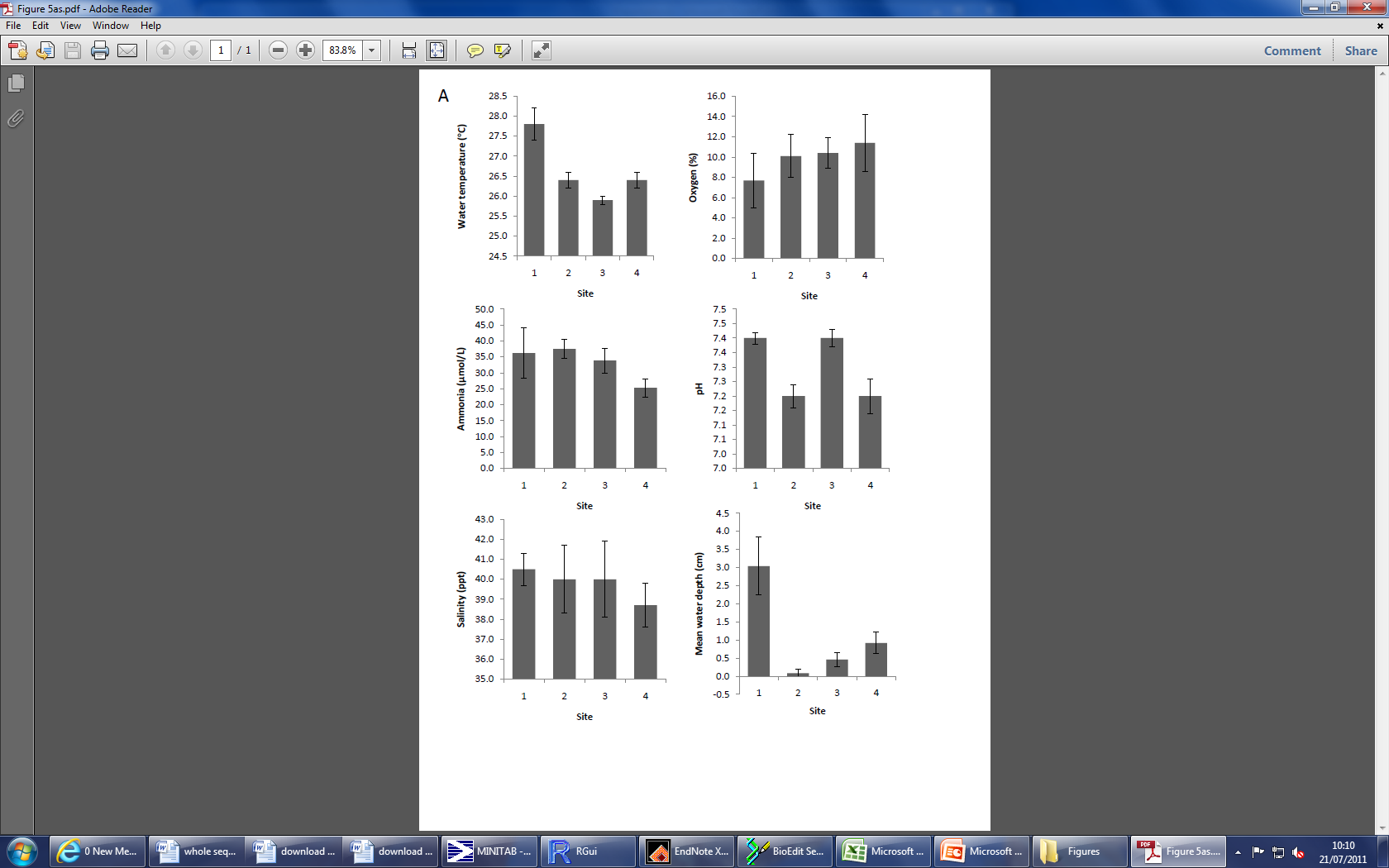
**

**Figure S3.** Mean (± 1 SE) water temperature, % oxygen, ammonia, pH, salinity and water depth of 4 sampling sites at Calabash Caye, Belize.
